# Long-Term Effects of Maternal Medication on Global Quality of Life Measured with SEQOL. Results from the Copenhagen Perinatal Birth Cohort 1959—61

**DOI:** 10.1100/tsw.2003.58

**Published:** 2003-08-18

**Authors:** Soren Ventegodt, Joav Merrick

**Affiliations:** The Quality of Life Research Center, Copenhagen K, Denmark

**Keywords:** birth cohort, longitudinal study, maternal health, child health, development, quality of life, maternal medication, Denmark

## Abstract

The Copenhagen Perinatal Birth Cohort 1959—61 is a prospective longitudinal perinatal study that included all deliveries (over 20 weeks gestation, birthweight over 250 g) that took place at the University Hospital (Rigshospitalet) in Copenhagen, Denmark during the period of September 21, 1959 to December 21, 1961 and used in this follow-up study to investigate the connection between maternal medication during pregnancy and the quality of life of the child 31 to 33 years later. The latest follow-up study from the cohort was performed in 1993 and 7,222 of the surviving children were identified (now aged between 31 and 33 years) and contacted with a nonanonymous questionnaire on several aspects of quality of life issues.There were 4,626 usable responses (f = 2,489, m = 2,131) corresponding to a response rate of 64.1%. Of the 12 groups of medication taking during pregnancy we found, before controlling (using multiple linear regression), that analgesics, chemotherapy, and psychopharmacologica showed links with the quality of life in the child 31 to 33 years later. Barbiturate use (95% was phenemal) showed significant connection to quality of life. After controlling for social and pregnancy factors there was no correlation between quality of life and medication taken by the mother during pregnancy. From this study it is concluded the fetal exposure to the drugs examined showed no measurable long-term effects on quality of life.

## INTRODUCTION

The potential risks stemming from fetal exposure to medication taken by the mother during pregnancy have been well researched[1,2]. Great efforts have been made to ensure that medications prescribed to pregnant women carry minimal risks to the unborn child.

However, in most cases the information on which drug safety agencies base risk assessment of adverse outcomes relates to outcome measures based on antenatal and perinatal examinations and rather less is known about possible long-term effects. The case could be that prenatal exposure to medication may have effects that only manifest themselves in adult life.

Long-term effects on intelligence deficits after *in utero* exposure to phenobarbital have been studied[3] and it was found that men exposed had significantly lower verbal intelligence scores (approximately 0.5 SD) than predicted, with an increased negative effect for those of lower socio-economic status those offspring from an "unwanted" pregnancy.

In this presentation we used a large birth cohort study sample in order to investigate the long-term effects on quality of life (QOL), when the mother was exposed to various medications during pregnancy.

## METHODS

### Population

The Copenhagen Perinatal Birth Cohort 1959–61[4,5,6,7] is a prospective longitudinal perinatal study that included all deliveries (over 20 weeks gestation, birthweight over 250 g) that took place at the University Hospital (Rigshospitalet) in Copenhagen, Denmark during the period of September 21, 1959 to December 21, 1961. The cohort consisted of 9,006 pregnant women who delivered 8,820 live born infants and 362 stillborn or late abortions over 250 g. 170 women gave birth to twins, three to triplets and the remainder were single births. The pregnant women were contacted and examined before delivery as early in pregnancy as possible. In order to evaluate and code the social, medical, and obstetrical information uniformly, all examinations were done by the same physician[4]. He also met with all women after delivery and coded information recorded by the women themselves during pregnancy and also the information describing the events in the delivery room[7].

The infants were examined by one of three pediatricians on the first and fifth days. Physical and neurological examinations were conducted. Upon discharge the mothers received a questionnaire related to child development during the first year, which they brought to the hospital at the age of 1 year for a physical reexamination (a total of 8,425 children survived 28 days)[5,6,7]. Follow-up physical examinations took place at 3 and 6 years with developmental recording done by the mothers between examinations. At a later stage all school health records were retrieved and coded by one physician[8]. More than 1,000 factors relating to pregnancy, birth, and child development were collected on each child resulting in numerous scientific publications over the last 40 years.

In 1993 a new follow-up study was performed and 7,222 of the surviving children were identified (now aged between 31 and 33 years) and contacted with a nonanonymous questionnaire. A written reminder to nonresponders was sent a month later resulting in 4,626 usable responses (f = 2,489, m = 2,131) corresponding to a response rate of 64.1%. The response rate for each individual question was typically a little lower.

## The SEQOL Questionnaire

The questionnaire used in the study, “Questionnaire for the Self Evaluation of Quality of Life” (SEQOL), contained 317 questions with 205 in an easy-to-answer multiple-choice format. The questionnaire was divided into the following sections: social information, lifestyle, illness, sexuality, self-perception, life perception, and eight series of questions measuring the quality of life[9].

The development of the questionnaire and progression of the study was guided by the following methodological demands for quantitative questionnaire-based quality of life research[9]:

1. A clear definition of the quality of life.
2. A philosophy of life based on the definition of point 1.
3. A theory that has this philosophy as its framework by
   a) deducing questions that are unambiguous, mutually exclusive, and together are fully exhaustive, and by
   b) establishing the relative weighting of each question.
4. A number of response options that might be given a quantitative interpretation on a fraction scale.
5. Technical checks in terms of reproducibility, sensitivity, well-scaledness, etc.
6. The survey must be meaningful to both researchers, respondents, and those who use the results.
7. An appreciation of the aesthetic dimension.

The present study followed these requirements[9] with the theoretical basis for quality of life measurement based upon the integrative quality of life theory[9]. It organises eight individual theories of quality of life into a spectrum ranging from subjective (self-evaluated) to objective (externally evaluated) quality of life and spanning a core of theories that consider quality of life as deriving from human nature or human existence itself (existential theories). These eight theories or dimensions of life quality were operationalized into eight rating scales, then grouped into three dimensions:

**I. Subjective Dimensions**

1. Immediate, self-experienced well-being
2. Satisfaction with life
3. Happiness

**II. Existential Dimensions**

4. Needs fulfillment
5. Subjective experience of objective temporal domains (family, work, leisure)
6. Subjective experience of objective spatial domains (satisfaction with social relationships)
7. Expression of life’s potentials

**III. Objective Dimension**

8. Objective factors (income, employment, education, etc.)

Eighty-five of the questions in the questionnaire were used to measure quality of life along these eight dimensions. A Likert-scale with five response options was symmetrically arranged around a neutral midpoint. As an example, well-being was measured by the question, “How are you feeling now?”, and the response options given were “very good,” “good,” “neither good nor poor,” “poor,” and “very poor.” The central and precisely worded mid-point (neither good nor poor), the response options symmetrically aligned up and down the scale (good, poor) and the use of the same amplifier (very) all combine to suggest that the five points on the scale may be considered equidistant.

If an underlying scale was selected ranging from 0 to 100%, from the worst imaginable to the best imaginable quality of life, the five response options may be reasonably positioned at 10, 30, 50, 70, and 90%. In other words, if a respondent checks “good,” his or her well-being is measured as 70%. In this manner, an approximated ratio scale was obtained, so that means could be computed and compared.

A weighted mean for the eight quality of life dimensions was computed by subjective and existential measures, respectively. The resulting overall measure was global (covers all aspects of life, not merely health-related aspects) and generic (not disease-related or intended for a specific category of patients).

Significance levels for the relationships between each variable and the measured quality of life were computed for the continuous variable using classical correlation and a modified regression[9], while in the case of the discrete variables, every group was tested individually against the rest of the sample HO: $\mu i = \mu non\text{-}i$ (that is, the null hypothesis that the mean quality of life for a particular group [for example, hormones taken by the mother] was significantly different from the mean quality of life for the rest of the population [for example, those that do not smoke]).

The questionnaire has been validated[9,10,11,12], and the measurement instruments (the rating scales) proved to be valid and sensitive to the same degree as commonly recognised international instruments. A 1-month and a 3-month test-retest for reproducibility showed correlation coefficients for the eight instruments ranging from 0.6 to 0.9. A qualitative assessment of the validity of the questionnaire was performed, in which 80% of the respondents indicated that the questionnaire items expressed all dimensions relating to their quality of life, 17% were in doubt, and 3% felt they did not express all dimensions, which was found to be acceptable.

## RESULTS

In this study quality of life was examined across the eight dimensions discussed above, ranging from the subjective to the objective, while information on medication *in utero* was taken from self-reported data and physician interview or notes, collected during the paediatric study.

Table 1 shows the connections between quality of life and the drugs with which the mother was treated during pregnancy. We found, before controlling (using multiple linear regression), that only analgesics, chemotherapy, and psychopharmacologica showed links with the quality of life in the child 31 to 33 years later. Barbiturate use (95% of the cases phenemal) showed no significant connection to quality of life.

After controlling for social and pregnancy factors there exists no correlation between quality of life and medication taken by the mother during pregnancy.

## DISCUSSION

It would seem from our study that *in utero* exposure to the medications seen in Table 1 had no long-term effects on quality of life. However, it is interesting to note that before controlling the

**TABLE 1**
**Quality of Life at 31 to 33 Years of Age vs. Mother's Drug Treatment during Pregnancy**

| Drug used in pregnancy | % | 1 M | 1 D | 2 M | 2 D | 3 M | 3 D | 4 M | 4 D | 5 M | 5 D | 6 M | 6 D | 7 M | 7 D | 8 M | 8 D | Overall QL (Weighted) | D | Test (p-value) |
|---|---|---|---|---|---|---|---|---|---|---|---|---|---|---|---|---|---|---|---|---|
| 1 None of these | 49.3 | 72.0 | 0.1 | 69.4 | 0.1 | 66.1 | 0.0 | 68.4 | 0.1 | 74.9 | 0.0 | 67.2 | 0.1 | 61.8 | 0.6 | 72.8 | -0.5 | 70.0 | -0.1 | |
| 2 Antihistimines | 12.1 | 72.4 | 0.7 | 70.2 | 1.2 | 67.3 | 1.8 | 69.0 | 1.0 | 75.4 | 0.7 | 67.4 | 0.5 | 62.1 | 1.2 | 75.6 | 3.4 | 71.4 | 1,8 | * 0,0069 |
| 3 Anti epileptic | 1.0 | 73.7 | 2.5 | 72.8 | 5.0 | 69.1 | 4.4 | 68.1 | -0.3 | 74.7 | -0.3 | 67.8 | 1.0 | 59.9 | -2.4 | 73.0 | -0.2 | 70.8 | 1,1 | 0.4411 |
| 4 Barbiturates | 10.5 | 72.6 | 0.9 | 71.0 | 2.4 | 66.6 | 0.7 | 69.0 | 1.0 | 75.3 | 0.5 | 67.7 | 0.9 | 60.9 | -0.8 | 73.7 | 0.8 | 70.7 | 0,8 | 0.3443 |
| 5 Psychopharma-cological | 9.2 | 70.1 | -2.5 | 68.1 | -1.9 | 65.1 | -1.5 | 67.7 | -0.9 | 74.6 | -0.4 | 66.2 | -1.3 | 60.1 | -2.2 | 72.3 | -1.1 | 69.1 | -1.4 | 0.0301 |
| 6 Analgesic | 3.7 | 71.1 | -1.1 | 68.0 | -2.0 | 65.5 | -1.0 | 66.5 | -2.6 | 72.6 | -3.0 | 65.0 | -3.1 | 58.1 | -5.3 | 69.6 | -4.8 | 67.8 | -3.2 | 0.0267 |
| 7 Antibiotics | 2.8 | 72.2 | 1.1 | 70.0 | 0.9 | 66.0 | -0.2 | 69.0 | 1.0 | 75.8 | 1.1 | 66.7 | -0.5 | 61.9 | 0.8 | 72.3 | -1.1 | 70.1 | 0.0 | 0.9479 |
| 8 Chemotherapy | 8.4 | 69.9 | -2.7 | 67.0 | -3.5 | 64.8 | -2.0 | 68.3 | -0.1 | 73.6 | -1.7 | 66.5 | -0.9 | 60.5 | -1.4 | 72.1 | -1.4 | 68.9 | -1.7 | 0.0496 |
| 9 Hormones - antihormones | 5.9 | 72.5 | 0.8 | 70.6 | 1.8 | 68.1 | 2.9 | 69.6 | 1.8 | 76.3 | 1.8 | 69.1 | 2.9 | 63.1 | 2.7 | 75.7 | 3.5 | 71.8 | 2,5 | * 0,0054 |
| 10 Diuretics | 14.5 | 72.5 | 0.9 | 67.7 | 0.6 | 67.2 | 1.5 | 68.5 | 0.3 | 75.1 | 0.2 | 67.6 | 0.7 | 61.5 | 0.2 | 73.8 | 1.0 | 70.6 | 0,8 | 0.1667 |
| 11 Other + vaccinations | 11.3 | 71.8 | -0.1 | 68.8 | -0.7 | 65.6 | -0.8 | 67.9 | -0.6 | 74.0 | -1.2 | 66.9 | -0.2 | 60.7 | -1.1 | 74.9 | 2.5 | 70.4 | 0,4 | 0.5397 |
| | 128.7 | 71.9 | | 69.4 | | 66.5 | | 68.4 | | 74.8 | | 67.1 | | 61.0 | | 73.3 | | 70.2 | | |

Columns: 1 – Immediate, self–experienced well–being; 2 – Life satisfaction; 3 – Happiness; 4 – Fulfillment of needs; 5 – Experience of objective, temporal domains (family, work, leisure); 6 – Experience of objective, spatial domains (self, others, world); 7 – Expression of life's potentials; 8 – Objective factors data, exposure to hormones or anti-hormones was linked to an increased quality of life. This is probably explained by the fact that hormone treatment during pregnancy, some 35 years ago, was associated with a better standard of care and a higher standard of living. Thus the slightly higher quality of life seen could probably be due to the socioeconomic factor.

It was interesting to note that mild analgesics, sometimes thought to be relatively harmless, have a negative influence on quality of life. It is likely that this finding was more a reflection on the mother's socioeconomic circumstances rather than the physical properties of the analgesic. Earlier research has demonstrated that the minor medical complaints, which typically necessitate the use of mild analgesic, are closely linked to quality of life[13,14]. Therefore, recourse to these drugs may in fact indicate a low quality of life of the mother and it may not be surprising to find that the reduced quality of life seen in the child 31 to 33 years later may be the result of social inheritance, in which the child grows up adopting the negative attitudes and behaviours of the mother.

## CONCLUSION

It is always advisable to be cautious of taking medicine during pregnancy, but in relation to the examined drugs we could not find any negative long-term effects on quality of life in later adult life.

## ACKNOWLEDGEMENTS

This paper reports results from the Danish Quality of Life Population Survey, the planning and execution of which would not have been possible without the aid of Dorte Loldrup Poulsen, Jørgen Hilden and the late Bengt Zachau-Christiansen. Funds were received from several Danish Foundations, including The 1991 Pharmacy, the Goodwill, the JL, E. Danielsens & Wife's, Emmerick Meyer's, the Frimodt-Heineken, the Family Hede Nielsen's, Petrus Andersen's, C.P. Frederiksen's and the Wedell-Wedellsborgs Foundations, and IMK Almene Fond. The research was approved by the Copenhagen Ethical Committee under numbers (KF) V 100.1762/90 and (KF) 01-502/93.

## References

1. Shehata, H.A., and Nelson-Piercy, C. (2001) Drugs in pregnancy. Drugs to avoid. *Best Pract. Res. Clin. Obstet. Gynaecol.* 15(6), 971-986.
2. Ornoy, A. The effects of alcohol and illicit drugs on the human embryo and fetus. *Isr. J. Psychiatry Relat. Sci.* 39(2), 120-132.
3. Reinisch, J.M., Sanders, S.A., Mortensen, A.L., and Rubin, D.B. (1995) In utero exposure to phenobarbital and intelligence deficits in adult men. *JAMA* 274, 1518-1525.
4. Villumsen, A.L. (1970) *Environmental Factors in Congenital Malformations. A Prospective Study of 9,006 Human Pregnancies.* FADL Publishers, Copenhagen.
5. Zachau-Christiansen, B. (1972) *The Influence of Prenatal and Perinatal Factors on Development during the First Year of Life with Special Reference to the Development of Signs of Cerebral Dysfunction. A Prospective Study of 9,006 Pregnancies.* Poul A. Andersen, Elsinore.
6. Zachau-Christiansen, B. and Ross, E.M. (1975) *Babies*: *Human Development during the First Year*. John Wiley, London.
7. Merrick, J., Merrick, Y., and Zachau-Christiansen, B. (1983). The Copenhagen Perinatal Cohort 1959–61. *Int. J. Rehab. Res.* **6**(1), 57–59.
8. Merrick, J, Teasdale, T.W., and Merrick, Y. (1983) School health screening of a birth cohort: A prospective longitudinal study. *Int. J. Rehab. Res.* **6**(4), 461–468.
9. Ventegodt, S. (1996) *Measuring the Quality of Life: From Theory to Practice.* Forskningscentrets Forlag, Copenhagen.
10. Lindholt, J.S., Ventegodt, S., and Henneberg, E.W. (2002) Development and validation of QOL5 for clinical databases. A short, global and generic questionnaire based on an integrated theory of life. *Eur. J. Surg.* **168,** 107–113.
11. Ventegodt, S., Hilden, J., and Merrick, J. (2003) Measuring the quality of life: A methodological framework. *Quality Life Res.*, submitted.
12. Ventegodt, S., Henneberg, E.W., Merrick, J., and Lindholt, J.S. (2003) Validation of two global and generic quality of life questionnaires for population screening: SCREENQOL and SEQOL. *TheScientificWorldJOURNAL* **3**, 412–421.
13. Ventegodt, S. (1996) *Quality of Life of 4,500 31-33 Year Olds*. Forskningscentrets Forlag, Copenhagen.
14. Ventegodt, S. (1995) *Quality of life in Denmark. Results from a Population Study*. Forskningscentrets Forlag, Copenhagen.

**BIOSKETCH**

**Søren Ventegodt, MD,** is the director of the the Quality of Life Research Center in Copenhagen, Denmark. He is also responsible for a research clinic for holistic medicine in Copenhagen and used as a popular speaker throughout Scandinavia. He has published numerous scientific and popular articles and a number of books on holistic medicine, quality of life, and quality of working life. His most important scientific contributions are the comprehensive SEQOL questionnaire, the very short QoL5 questionnaire, the integrated QOL theory, the holistic process theory, the life mission theory, and the Danish Quality of Life Research Survey, 1991–94 in cooperation with the University Hospital of Copenhagen and the late pediatric professor Bengt Zachau-Christiansen. Website: www.livskvalitet.org/

**Joav Merrick, MD, DMSc,** is professor of child health and human development affiliated with the Zusman Child Development Center and Division of Community Health at the Ben Gurion University, Beer-Sheva, Israel and presently the medical director of the Division for Mental Retardation, Ministry of Social Affairs, Jerusalem and the director of the National Institute of Child Health and Human Development. He has authored and edited numerous publications in the fields of child and human development, rehabilitation, intellectual disability, disability, health, welfare, abuse, advocacy and prevention. Dr. Merrick received the Peter Sabroe Child Award for outstanding work on behalf of Danish Children in 1985 and the International LEGO-Prize ("The Children's Nobel Prize") for an extraordinary contribution toward improvement in child welfare and well-being in 1987. Website: www.nichd–israel.com
